# Changes in Morphology and Presence of Pinopodes in Endometrial Cells during the Luteal Phase in Women with Infertility Problems: A Pilot Study

**DOI:** 10.3390/medicina54050069

**Published:** 2018-10-10

**Authors:** Marina Aunapuu, Piret Kibur, Tõnu Järveots, Andres Arend

**Affiliations:** 1 Department of Anatomy, University of Tartu, Ravila 19, 50411 Tartu, Estonia; piret.kibur@kliinikum.ee (P.K.); andres.arend@ut.ee (A.A.); 2 Department of Morphology, Estonian University of Life Sciences, Fr. Kreutzwaldi 62, 51014, Tartu, Estonia; tonu.jarveots@emu.ee

**Keywords:** endometrium, ciliated cells, secretory cells, pinopodes, infertility

## Abstract

*Objective:* To investigate morphological changes in the endometrial epithelial cells of patients with infertility problems. *Materials and methods:* Endometrial biopsies were obtained from 10 women who have undergone several unsuccessful in vitro fertilisation (IVF) procedures. Endometrial biopsies were performed between luteinizing hormone surge days LH+6 to +10 of the natural menstrual cycle. Each sample was divided into three parts, which were processed for histological, transmission (TEM), and scanning electron microscopy (SEM) investigations. *Results:* Histological investigations demonstrated significant alterations in the apical part of epithelial cells of one patient; in four patients, the gland maturity was low, not matching the cycle day, and thus a phase lag had developed. By TEM examination, we ascertained changes in secretory and ciliated cells in three patients (decreased amount or missing microvilli, irregular cilia in ciliated cells). SEM examination found pinopodes in five patients: three samples contained fully developed pinopodes—larger and completely smooth, with only some wrinkles; one sample contained regressing small pinopodes, with wrinkled surfaces; and one sample had both developed and regressing pinopodes. *Conclusions:* To conclude, our study shows that the endometrium of patients with poor IVF outcome has either significant changes in the morphology or the endometrial maturation is inhibited and a phase lag often develops. Our study shows that endometrial pinopodes are found throughout the mid-luteal phase up to day LH+10.

## 1. Introduction

Infertility is a condition that affects a couple and is defined as the lack of conception after an arbitrary period of 12 months with regular sexual intercourse and without using any contraception. Europe is the continent with the lowest fertility [1], and infertility is a common medical problem present in about 10% of couples of reproductive age in most European countries. It is estimated that 75 million couples worldwide have problems with having children. However, merely every second infertile couple seeks medical advice [2]. Both the female’s and male’s medical problems may lead to a couple’s infertility. In about 80% of cases, the medical cause for infertility can be identified, and in about 20% of couples, the reason for infertility remains unexplained [3]. About 70% of the cases of infertility in couples have been caused by the female factor, 25% by the male factor, and in 5% of the couples, infertility problems are seen in both partners [3]. Female infertility is mainly caused by tubal factor infertility, impaired endometrial function, and endocrine dysfunctions.

Among medical treatments, in vitro fertilization (IVF) is the most widely used method in the treatment of infertility. IVF pregnancy results depend on the quality of the endometrium, i.e., its receptivity [4]. Unfortunately, IVF pregnancy results are low and the treatment is expensive and associated with various medical risks for both the mother and the child. The progress in IVF has so far been focused on the improvement of the developmental potential of in vitro embryos, while the possibilities for the evaluation of endometrium receptivity have remained relatively limited. Although there are well-characterised morphological and molecular markers of implantation, the complete dynamics of the process as well as the importance of each and every marker is still vague. One precondition for the successful implantation of an embryo is the presence of pinopodes on the receptive endometrium [5,6]. By the time of implantation, luminal epithelial cells protrude their apical plasma membranes and form pinopodes [5,7]. The formation of pinopodes is progesterone-dependent [8]. Although the presence of endometrial pinopodes is generally considered as an evident manifestation of a receptive endometrium, the direct involvement of pinopodes in embryo–endometrial interactions has not yet been proven. Nevertheless, an association between the density of pinopodes on apical membranes of luminal epithelial cells and the effectiveness of in vitro fertilization-embryo transfer (IVF-ET) has been shown [9]. Scanning electron microscopy (SEM) studies have shown that pinopodes appear between the 20th and the 22nd day of the menstrual cycle and are able to exist for two days [9]. Still, the importance of pinopodes in implantation is questioned, since the presence of pinopodes has been observed during the entire secretion phase of the menstrual cycle and even after the implantation period up to the 11th week of gestation [10]. However, in recent years, several studies have been published supporting the pinopodes as reliable markers of endometrial receptivity [11,12].

The aim of our study was to investigate the changes in endometrial morphology of infertile women and examine the apical surface of epithelial cells.

## 2. Materials and Methods

### 2.1. Human Subjects

Endometrial biopsies from 10 patients with infertility problems were collected (Nova Vita Clinic, Tallinn, Estonia). The age of the patients was from 28 to 40 years (Table 1), and they all had undergone several unsuccessful IVF procedures. Endometrial biopsies were obtained from informed women before their participation in the study. The protocol for the research project no. 7301 has been approved by the Ethics Review Committee on the Human Research of the University of Tartu (Protocol no. 161/18, June 18, 2007) and it is in accordance with the Declaration of Helsinki (1975). The biopsy was taken under narcosis between day LH+6 to +10 in a natural menstrual cycle, where LH = 0 was the day of the serum LH surge (Table 1). The estradiol and progesterone blood serum levels of patients were within normal values for the luteal phase (except the progesterone levels of patient nos. 2 and 10). Endometrial thickness was determined by sonography (Table 2).

### 2.2. Histology

Each sample was divided into different specimens and immediately fixed. One specimen was processed for light microscopy investigation, the second specimen for transmission electron microscopy (TEM), and the third specimen for SEM investigation. Specimens for light microscopy were fixed in 10% formalin for 12 h and embedded in paraffin with a vacuum infiltration processor (Tissue-Tek^®^ VIP^TM^ 5 Jr, Sakura Finetek Europe B. V., Zoeterwoude, The Netherlands). Specimens were cut with a microtome (Ergostar HM 200, Microm, Germany) into 4-µm-thick sections and stained using hematoxylin–eosin (H&E) for the general orientation of sections. Slides were observed and photographed by a Zeiss Axiophot 2 microscope (Carl Zeiss AG, Jena, Germany).

### 2.3. Transmission Electron Microscopy

For TEM investigations, specimens were fixed in 2.5% glutaraldehyde solution (Sigma-Aldrich Chemie GmbH, Germany) buffered with sodium cacodylate buffer (Sigma-Aldrich Chemie GmbH, Germany) at pH = 7.4 for two hours at 4 °C. The samples were postfixed for 1 h in 1% osmium tetroxide solution (Agar Scientific, Stansted, UK) at the same temperature and pH, dehydrated at increasing concentrations of ethanol (50, 70, 90, 96, 99.5%), in an acetone series and embedded in Epon-812 (Fluka Chemie AG, Buchs, Switzerland) according to standard methods. Semithin sections were stained with methylene blue-azur II to select the region of interest for the following procedures. The semithin sections were analysed using a Zeiss Axiophot 2 microscope (Zeiss, Germany). The ultrathin (80 nm) sections were cut on the Reichert Om U3 ultratome with a diamond knife (Diatome Ltd, Biel/Bienne, Switzerland). Sections were mounted on copper grids of mesh size 200 (Sigma-Aldrich Chemie GmbH, Germany) with Perfect Loop (Diatome Ltd., Biel/Bienne, Switzerland) and stained with uranyl acetate (Agar Scientific, Stansted, UK) and lead citrate (Agar Scientific, Stansted, UK) according to standard methods. For TEM, a Philips Tecnai-10 with camera Mega View II was used for viewing and photographing.

### 2.4. Scanning Electron Microscopy

For SEM examinations, samples were fixed in 2.5% glutaraldehyde solution buffered with sodium cacodylate buffer at pH = 7.4 for two hours. The specimens were dehydrated in increasing concentrations of ethanol (50, 70, 96, and 99.5%) in an acetone series and dried using a critical point drier. After drying, the samples were mounted on an aluminium stub using silver paint. The samples were then introduced into the chamber of the sputter coater and coated with gold. For SEM, a LEO-1430 VPSE was used for viewing and photographing. Ciliated and secretory cells were counted at 4050× magnification in five viewing fields per sample, and the proportion between these two cell types (in percentages) was calculated. In addition, pinopodes were identified and recorded as fully developed pinopodes or regressing pinopodes. The number of SEM images counted for each patient was 20.

## 3. Results

### 3.1. Histology

**Figure 1:**
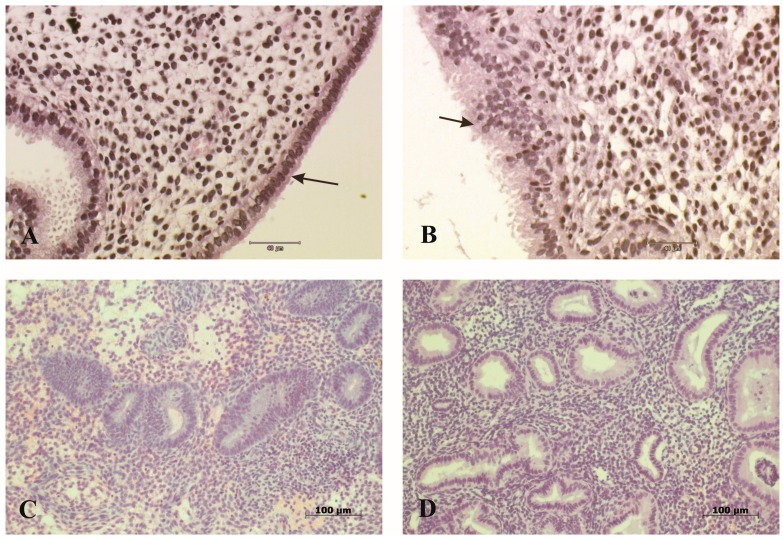
Histology: (**A**) Regular simple columnar epithelium of the endometrium of patient no. 1 (arrow); H&E. (**B**) Impaired endometrial epithelium (arrow) found in patient no. 3; H&E. (**C**) Endometrium with equal (1:1) gland–stroma ratio of patient no. 4; H&E. (**D**) Endometrium with prevailing connective tissue stroma (2:1) in patient no. 6; H&E.

The endometrial luminal surface of the biopsy sample was identified in all histology slides. Simple columnar epithelium of the endometrium appeared to be normal and contained ciliated and nonciliated epithelial cells in eight patients (Figure 1A), while in two patients, the apical part of epithelial cells was damaged (Figure 1B). In one patient, epithelial cells contained glycogen inclusions. The investigation revealed that in four patients, the morphological picture and the endometrial gland maturity did not coincide with the cycle day. The endometrial gland maturity of these patients was low, and, correspondingly, a phase lag had developed (Table 1). The gland–stroma ratio in the endometrium was equal (1:1) in the four investigations (Figure 1C); in two cases, the glands were prevailing, and in four cases, the connective tissue stroma was prevailing (Figure 1D, Table 2). The endometrial stroma of the examined samples contained a large amount of connective tissue cells and a smaller amount of lymphocytes and neutrophilic granulocytes. The endometrial biopsies of six patients contained well-expressed spiral arteries, while no spiral arteries were found in biopsies of four patients (Table 2).

### 3.2. TEM

**Figure 2:**
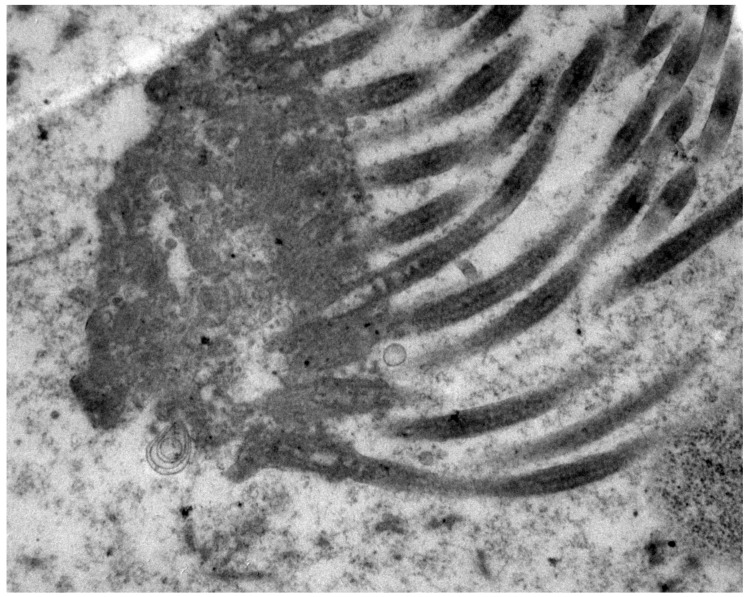
Ultrastructure of ciliated cell with regular cilia (patient no. 5); TEM 17000×.

In ultrastructure investigations, we found changes in secretory and ciliated cells in three patients. The apical surface of secretory epithelial cells had the presence of a small amount or absence of microvilli, and ciliated cells had short and partly coalesced cilia. In six patients, no changes in secretory and ciliated cells were found (Figure 2). No changes were found in the nuclei and endoplasmic reticulum of epithelial cells.

### 3.3. SEM

**Figure 3:**
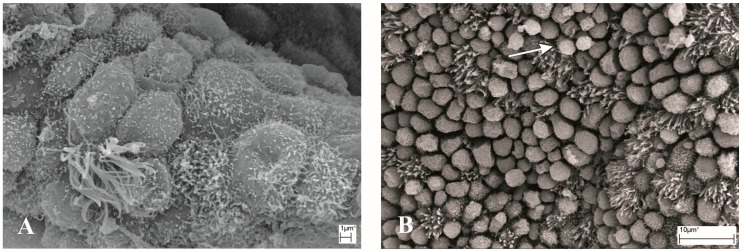

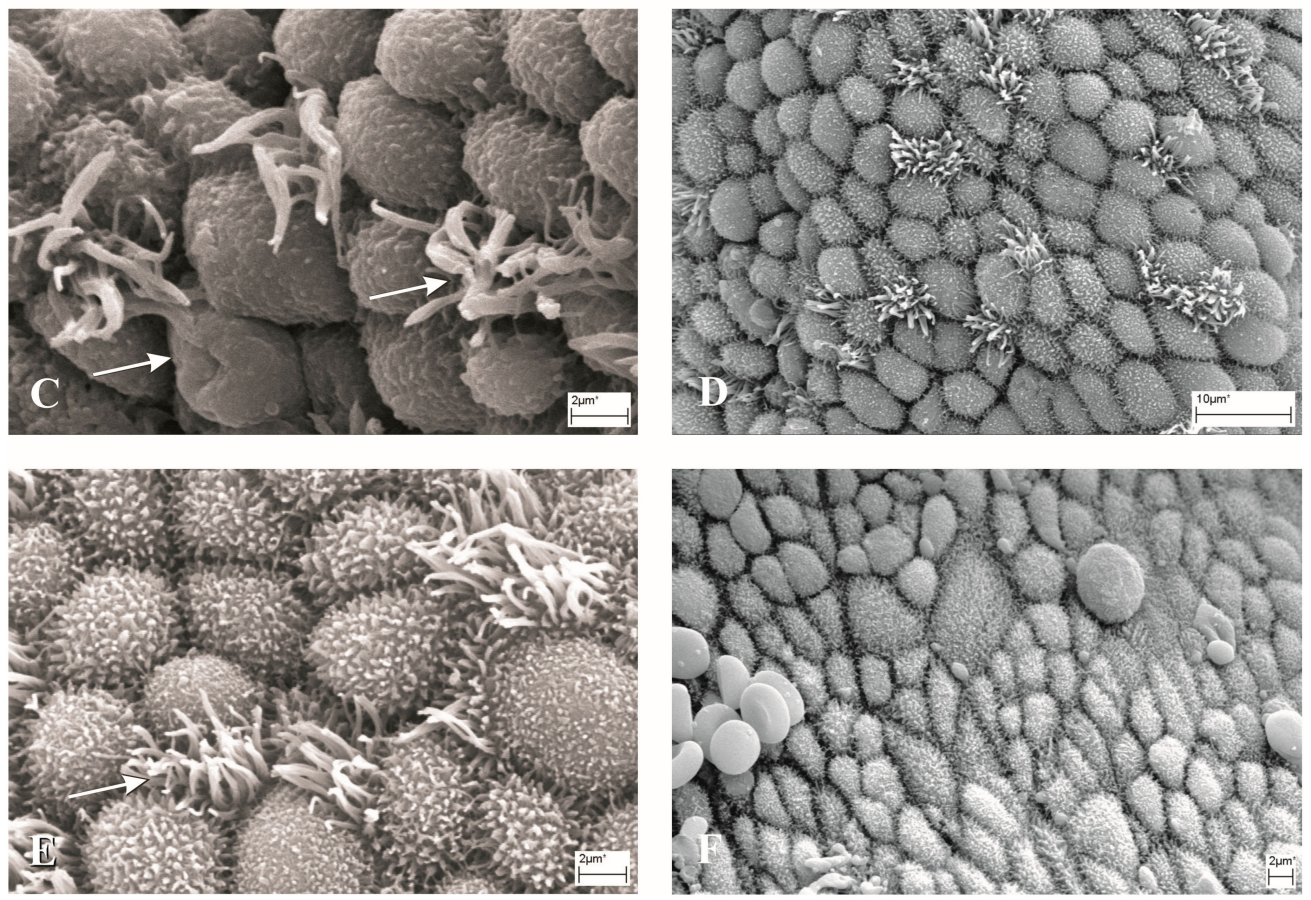
SEM: (**A**) “Bare” endometrial cells with few cilia and microvilli (patient no. 1). (**B**) Regressing small pinopodes with wrinkled surface (arrow, patient no. 2). (**C**) “Bare” endometrial cells with few cilia (arrows, patient no. 5). (**D**) Normal endometrial secretory epithelial cells (patient no. 8). (**E**) Normal endometrial secretory epithelial cells (arrow, patient no. 9). (**F**) Epithelial cells with short microvilli on the apical surface (patient no. 10).

The secretory and ciliated cells of uterine glands were studied by the SEM method (Figure 3). Although the number of secretory cells dominated over ciliated cells in all samples, in two patients, the proportion between these two cell types was relatively equal (Table 2). In six patient samples, SEM investigation showed normal endometrial secretory epithelial cells (Figure 3D,E). Secretory cells contained regular and short microvilli on the apical surface. Secretory epithelial cells of four patients had a small amount of or no microvilli, and thus the cells were “bare” (Figure 3A,C). In samples of six patients, long, regular cilia on the apical surface of ciliated cells were seen (Figure 3E).

Pinopodes, essential components for embryonic implantation, were present in five samples (Figure 3B, Table 2) and their amount in biopsies was varying. Three samples contained fully developed pinopodes—large and completely smooth with only some wrinkles; one sample contained regressing small pinopodes, with wrinkled surfaces; and one sample contained both fully developed and regressing pinopodes.

## 4. Discussion

Embryo implantation is dependent on both embryo and endometrium quality. This study shows that in the majority of 10 infertile women, the endometrium had low quality.

Uterine mucosa development is a complicated physiological process involving the remodelling of tissue properties with the aim to make it receptive to embryo implantation. Traditional techniques for studying endometrial quality are ultrasonography and histology. Endometrial sonography is a noninvasive method safe to be used during the IVF procedure. Endometrial biopsy is an invasive procedure, which can be performed only during the menstrual cycle [13] before the IVF cycle. Endometrial histological evaluation originates from a pioneering study by Noyes et al. [14] and is used to date the stages of epithelial and stromal development. The most common pathology in histological analysis of endometrial biopsy is the delayed secretion phase, where endometrial maturation is at least two days behind the ovarian cycle. This out-of-phase endometrial histopathology is usually caused by failure in the secretion and/or function of progesterone at the secretory phase. It is known that thickening of endometrial stroma takes place during the cycle, and at the end of the cycle, there is a change in the gland–stroma ratio in favour of the former. In our study, we found such a result in two patients. In four patients, the gland–stroma ratio was 1:1, which shows that the functional layer of their endometrium had not yet fully developed, and the morphological picture did not correspond to the cycle day. The second characteristic of endometrium maturity is the presence of spiral arteries in the functional layer. Spiral arteries grow together with the endometrium, being the characteristic structure of the functional layer. In biopsy samples studied by us, spiral arteries were distinguishable in five patients. Biopsy samples of four patients did not contain any spiral arteries and SEM investigation of these patients demonstrated pinopodes, considered as putative indicators of receptivity, only in one patient (Table 2). To complement histological studies, detection of putative markers of endometrial receptivity, such as integrins and mucins, have gained significant attention in the last decades. However, the expression of these markers does not seem to be synchronous with the formation of pinopodes [15]. In our preliminary immunohistochemical studies on integrin β3 and mucin 1 (MUC1) in the endometrium of infertile women, we found no significant differences from the control group values (unpublished data). Light microscopy investigations are insufficient for the study of the receptive endometrium, since important information can be added from ultrastructure investigations. Both TEM and SEM have been used for investigating endometrial tissue [5,12,16,17,18]. The endometrial epithelium consists of the secretory and the ciliated cells, and the apical surface of these cells can be clearly observed in SEM examination. In biopsy samples examined by us, secretory cells in three patients had no microvilli, a condition possibly produced by hormonal dysregulation. Histological and SEM investigation [5,6,7,12] have shown the presence of pinopodes on the apical plasma membrane of secretory cells; these structures were visible in five samples examined by us. However, it is surprising that, despite extensive research, the cellular function of pinopodes in humans it is still not known, and researchers have no uniform opinion about their function, although it is thought that pinopodes participate in implantation [6,7,11]. At the same time, opposite views have also been expressed, claiming that the role of pinopodes in implantation is not particularly important. Pinopode duration in the endometrium of different mammalian species has been reported to range from hours to weeks [7,10,19]; in particular, the human duration is extremely variable. Some researchers have shown that pinopodes persist for less than 48 h [7,20] during the mid-luteal phase; others have demonstrated that pinopodes are present shortly after ovulation and persist to the end of the luteal phase [10]. In the samples examined by us, pinopodes were visible from day LH+6 up to day LH+10, while on day LH+10, pinopodes were found in one patient out of four. Although most endometrial biopsies were not normal, our results indicate that pinopodes exist for approximately five days. This result corresponds to the morphological picture of the patients, which demonstrated a phase lag as compared to the cycle day. Proceeding from the opinion that pinopodes are extremely important for embryo implantation, preliminary conclusions can be made as to why many IVF procedures do not provide positive results. The endometrium development in infertile patients is slower, and often a phase lag occurs, which does not reflect the actual situation of the endometrium. Considering this lag phase, it is possible that a more successful outcome may be achieved if embryo transfer is delayed by one or a few days. However, different time schemes may need to be developed for procedures in the case of these patients where IVF has failed repeatedly. Considering the small number of patients in our study, a study on a larger scale is needed before any comprehensive conclusions can be made.

## 5. Conclusions

Our study shows that the endometrium of patients with poor IVF outcome has either significant changes in morphology, or the endometrial maturation is inhibited and often a phase lag develops, which fails to reflect the actual situation of the endometrium. Endometrial pinopodes were found during the mid-luteal phase from day LH+6 up to day LH+10, although on the latter day, pinopodes were seen only in one patient out of four.

## Figures and Tables

**Table 1:** Patients’ characteristics.

Patient No.	Age (Years)	Previous IVF Failure	LH+	Phase Lag	Estradiol (pmol/L)	Progesterone (nmol/L)	Testosterone (nmol/L)
1	29	1	6	+	756	53.7	1.45
2	36	1	6	−	727	≤0.64	≤0.49
3	38	1	7	−	474	25.8	2.8
4	32	1	8	+	822	66.5	1.96
5	31	2	9	−	602	33.4	1.78
6	30	1	10	−	437	30.2	2.58
7	40	1	10	−	536	38.2	0.87
8	28	1	10	+	492	17.8	2.28
9	38	2	10	+	709	49.3	0.91
10	34	2	6	+	148	3.21	1.35

IVF—in vitro fertilization, LH+—days after luteinizing hormone surge, phase lag +—phase lag determined, phase lag −—no phase lag determined.

**Table 2:** Results of sonographical and morphological investigations.

Patient No.	Endom. Thickness (mm)	Gland-to-Stroma Ratio	Spiral Arteries	SEM Investigations		
				Ciliated Cells (%)	Secretory Cells (%)	Pinopodes/Type
1	9.8	1:2	−	47.1	52.9	0
2	6.9	1:2	+	49.4	50.6	14F/10R
3	16.4	2:1	+	12.9	87.1	18/F
4	12.8	1:1	+	28.1	71.9	24/F
5	11.5	1:1	+	8.0	92.0	14/R
6	7.9	2:1	+	30.7	69.3	8/F
7	8.3	1:1	+	19.3	80.7	0
8	10.4	1:1	−	24.8	75.2	0
9	11	1:2	−	23.1	76.9	0
10	11.6	1:2	−	19.0	81.0	0

Endom. Thickness—endometrial thickness; + presence of spiral arteries in the endometrium; − spiral arteries were not found in the endometrium; F—fully developed pinopodes; R—regressing pinopodes.
